# A bacteriocin-based treatment option for *Staphylococcus haemolyticus* biofilms

**DOI:** 10.1038/s41598-021-93158-z

**Published:** 2021-07-06

**Authors:** Christian Kranjec, Sofie S. Kristensen, Karolina T. Bartkiewicz, Mikkel Brønner, Jorunn P. Cavanagh, Aparna Srikantam, Geir Mathiesen, Dzung B. Diep

**Affiliations:** 1 Faculty of Chemistry, Biotechnology and Food Science, Norwegian University of Life Sciences, Ås, Norway; 2 Pediatric Infections Group, Department of Pediatrics, University Hospital of North Norway, Tromsö, Norway; 3 Pediatric Infections Group, Department of Clinical Medicine, UiT the Arctic University of Norway, Tromsö, Norway; 4 Blue Peter Public Health and Research Centre, LEPRA Society, Hyderabad, India

**Keywords:** Antimicrobials, Biofilms, Pathogens, Molecular biology

## Abstract

Bacteriocins are ribosomally-synthesized antimicrobial peptides, showing great potential as novel treatment options for multidrug-resistant pathogens. In this study, we designed a novel hybrid bacteriocin, Hybrid 1 (H1), by combing the N-terminal part and the C-terminal part of the related bacteriocins enterocin K1 (K1) and enterocin EJ97 (EJ97), respectively. Like the parental bacteriocins, H1 used the membrane-bound protease RseP as receptor, however, it differed from the others in the inhibition spectrum. Most notably, H1 showed a superior antimicrobial effect towards *Staphylococcus haemolyticus*—an important nosocomial pathogen. To avoid strain-dependency, we further evaluated H1 against 27 clinical and commensal *S. haemolyticus* strains, with H1 indeed showing high activity towards all strains. To curtail the rise of resistant mutants and further explore the potential of H1 as a therapeutic agent, we designed a bacteriocin-based formulation where H1 was used in combination with the broad-spectrum bacteriocins micrococcin P1 and garvicin KS. Unlike the individual bacteriocins, the three-component combination was highly effective against planktonic cells and completely eradicated biofilm-associated *S. haemolyticus* cells in vitro. Most importantly, the formulation efficiently prevented development of resistant mutants as well. These findings indicate the potential of a bacteriocins-based formulation as a treatment option for *S. haemolyticus.*

## Introduction

Staphylococci are a diverse genus of Gram-positive bacteria, commonly found in the microbiota of skin and mucosal membranes of humans. Generally, staphylococci are divided into two groups: coagulase-negative (CoNS) and coagulase-positive staphylococci (CoPS), depending on the production of the clotting enzyme coagulase. For decades, the CoPS *Staphylococcus aureus* has been recognized as an important human pathogen, while CoNS have been considered commensal and regarded as mere contaminants when found in samples from infections^1,2^. Today, CoNS are recognized as major opportunistic nosocomial pathogens, particularly causing infections in immunocompromised patients and patients with indwelling medical devices^3,4^. Among CoNS, *S. haemolyticus* is receiving increased attention as a nonconical pathogen, being the second most frequently isolated CoNS in clinical settings^1,5^.

Compared to the more virulent *S. aureus*, few virulence characteristics have been determined as crucial for *S. haemolyticus* infections. One of these is its multidrug-resistant (MDR) phenotype^6–8^. Clinical isolates of *S. haemolyticus* are ranked as the most antibiotic-resistant CoNS, and they have frequently been reported as resistant to last-line antibiotics such as glycopeptides, making treatment options limited^1,9,10^. A variety of mechanisms contribute to the acquisition of antibiotic resistance. Notably, the *S. haemolyticus* genomes contain multiple insertions and single nucleotide polymorphisms (SNPs), allowing frequent genetic rearrangements^3,8^. The extreme genome plasticity likely allows *S. haemolyticus* to acquire antibiotic-resistant genes from its environment, often resulting in MDR hospital-adapted clones^8^. Equally significant is the horizontal gene transfer of antibiotic-resistance genes from *S. haemolyticus* to *S. aureus* strains observed in hospital outbreaks of methicillin-resistant *S. aureus* (MRSA), indicating that *S. haemolyticus* may act as a reservoir for resistance genes in hospitals^11–13^.

The ability to form biofilms and adhere to medical devices is another crutial virulence factor in *S. haemolyticus* infections^14^. Biofilm formation allows microbial adhesion to biotic and abiotic surfaces, as well as shielding the bacteria from the host immune response and antibiotics^15^. Biofilm formation on indwelling medical devices is a major concern, as the increased antibiotic resistance makes infections more difficult to treat, often leaving surgical removal of the device as the only option^14,15^. There is limited knowledge about the molecular mechanisms underlying the formation of *S. haemolyticus* biofilm. However, their primary importance in infections is evident. Compared to the polysaccharide biofilms formed by *S. epidermidis, S. haemolyticus* forms biofilms mainly composed of proteins and DNA^6^. The ability to form biofilms combined with their high degree of MDR makes infections caused by *S. haemolyticus* increasingly challenging to combat. Due to their ability to form biofilms, persist and thrive in the hospital environment^16^, *S. haemolyticus* strains are emerging as a severe nosocomial threat.

To better control the increasing number of MDR pathogens, novel treatment options are needed. In this context, bacteriocins are receiving increased scientific and medical interest. Bacteriocins are small ribosomally synthesized antimicrobial peptides which are produced by bacteria to compete with other bacterial species for nutrients and ecological niches. Bacteriocins are generally believed to be produced by most bacteria, with the *Staphylococcus* genus being no exception. Several bacteriocins produced by staphylococci show prominent anti-staphylococcal and bacteriolytic activities^17^.

Compared with traditional antibiotics, bacteriocins have several advantages, including low toxicity (particularly those expressed by lactc acid bacteria (LAB)), the ability to be bioengineered and high efficacy^18^. The high potency of bacteriocins has been demonstrated both in vitro and in vivo. Notably, bacteriocins exhibit high activity towards several clinically important species, including *Streptococcus pneumonia*^19^*,* MRSA^17,20^, *Clostridium difficile*^21,22^ and vancomycin resistant enterococci (VRE)^23,24^, thereby underlining the potential of bacteriocins as antimicrobial therapeutics. More importantly, bacteriocins have different modes of action than traditional antibiotics, making them active against MDR pathogens^18^.

Leaderless bacteriocins are synthesized without an N-terminal leader sequence and contain no post-translational modifications. This, along with their relatively small size (30–50 amino acids), makes them ideal for synthetic production. Enterocins K1 and EJ97 are two leaderless bacteriocins with potent and specific activity towards enterococcal species through an interaction with the membrane-bound site-2-protease RseP (Regulator of Sigma-E Protease)^23^. RseP is a virulence factor^25,26^ involved in the stress response and processing of signaling peptides in several bacteria^27–30^, thus serving as a potential antimicrobial target. It is proposed that EJ97 and K1 interact specifically with RseP to create pores in the cell membrane, thereby leading to disrupted membrane integrity and cell death^23^. In the present study, we designed a novel hybrid bacteriocin designated Hybrid 1 (H1), which is composed of the N-terminal half of Enterocin K1 and the C-terminal half of Enterocin EJ97^23,31^. Here we show that RseP serves as the receptor for H1 and that the hybrid bacteriocin has superior antimicrobial properties towards clinical *S. haemolyticus* isolates when compared with the parental bacteriocins*.* Moreover, we demonstrate that H1 acts synergistically with the broad-spectrum bacteriocins garvicin KS and micrococcin P1, not only to kill planktonic and biofilm-associated *S. haemolyticus* cells but also to prevent resistance development.

## Results

### The inhibition spectrum of the novel hybrid bacteriocin H1

**Figure 1:**
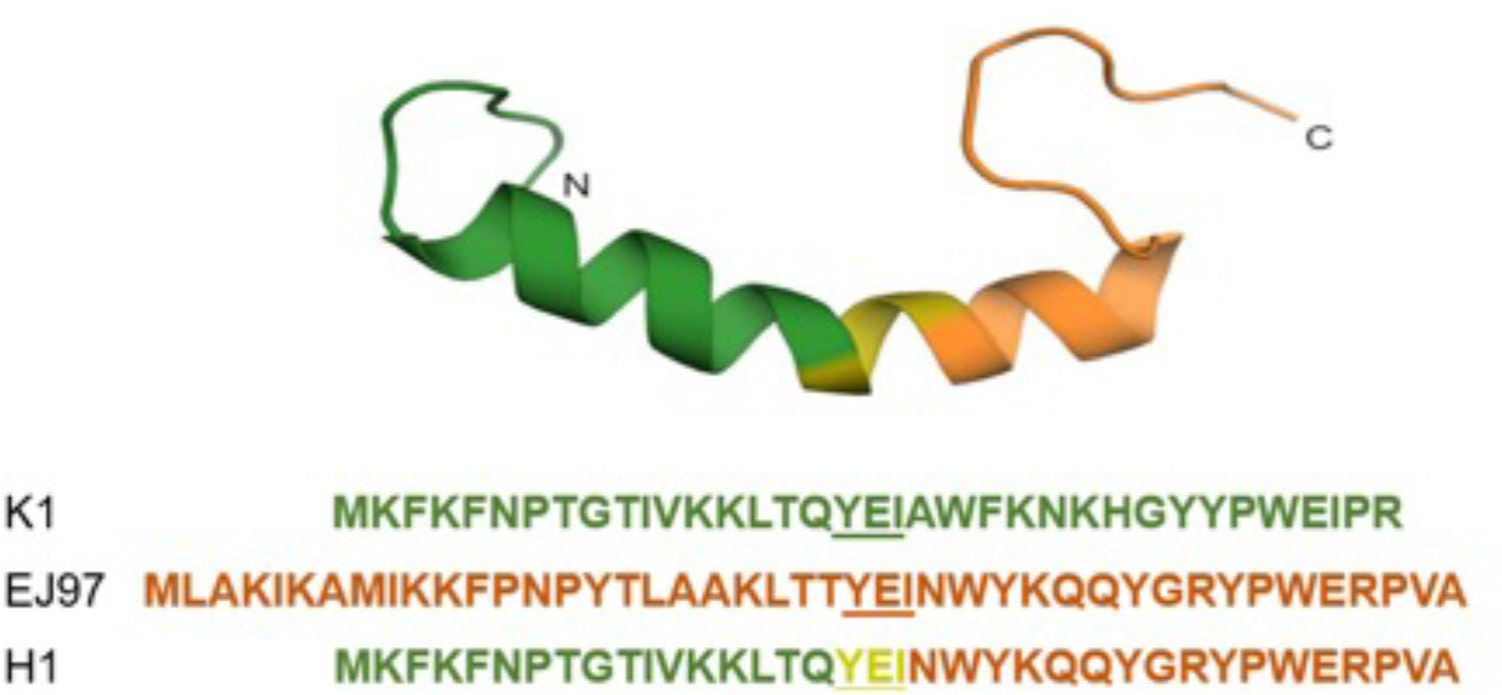
Predicted Structure and amino acid sequence of the hybrid bacteriocin and its parental bacteriocins. The predicted structure of the hybrid bacteriocin 1 (H1), formed by the N-terminal part of K1 (green) and the C-terminal part of EJ97 (orange). The central tripeptide sequence (YEI—underlined) in K1 and EJ97 serves as the joining segment in the fusion protein. The structure of H1 was modeled based on the NMR structural data of K1^23^ using the structure prediction tool Swiss model^35^.

Previous studies have indicated that the generation of hybrid bacteriocins can be an effective way to create novel antimicrobials with new and/or improved properties^32–34^. Inspired by these works we created a hybrid bacteriocin, called H1, formed by two sequence related enterocins: K1 and EJ97, the former being most active against *E. faecium* while the latter being most active against *E. faecalis*. H1 contains the N-terminal half of K1 and the C-terminal half of EJ97, sequence and predicted structure^23,35^, guided by the NMR data of K1, is shown in Fig. 1. When testing the antimicrobial activity against a panel of 50 bacteria from different genera and species (Table S1), the hybrid bacteriocin displayed good activity against selected species within the genera *Staphylococcus* and *Streptococcus*. Within the former genus, it is interesting to note that H1 displayed increased activity against *S. epidermidis* and *S. haemolyticus,* compared with the parental bacteriocins. In addition, all three bacteriocins showed weak or no effect on *S. aureus*, *S. arlettae* and *S. simulans*; whereas EJ97 and H1 displayed a comparable antimicrobial effect against *S. hominis*. Similarly, among streptococci, H1 showed increased activity against *S. thermophilus* and *S. uberis,* but no activity against *S. dysgalactiae*. None of the bacteriocins had activity against Gram-negative bacteria (Table S1). Because of the superior effect of H1 against the important human pathogen *S. haemolyticus*, we focused further on the activity of H1 on this species.

To examine whether the activity of H1 was strain-depedent, a collection of 27 clinical and commensal *S. haemolyticus* strains derived from blood, urine and wound-associated infections were tested (Table S2). Using the spot-on-lawn assay, H1 was found to have better activity (i.e., larger inhibition zones) for 74% of the tested *S. haemolyticus* strains compared to K1 or EJ97 (Table 1). However, development of resistant colonies was observed within the inhibition zones for all 27 strains after a 24 h incubation (Table 1). Using the microtiter plate assay to follow the antimicrobial dynamics, H1 had minimum inhibition concentrations (MIC_50_) in the range 0.1–0.78 mg/ml against the different *S. haemolyticus* strains after 5 h of incubation (Table S2) but resistance to H1 was progressively observed after 24 and 48 h incubation (data not shown).

**Table 1:** Inhibition spectrum of K1, EJ97 and H1 as assessed by the spot-on-lawn assay against 27 *Staphylococcus haemolyticus* strains.

*S. haemolyticus *strains	Bacteriocins (*)		
	K1	EJ97	H1
4068**	–	++*	+++*
4069**	–	++*	+++*
4070**	–	++*	+++*
4071**	–	++*	+++*
4072**	–	++*	+++*
4073**	–	++*	+++*
7067_4_56	–	++*	+++*
7068_7_63	–	++*	+++*
7067_4_66	–	++*	+++*
7067_4_49	–	++*	+++*
7067_4_84	–	++*	+++*
7067_4_67	–	++*	+++*
7067_4_71	–	+++*	+++*
7067_4_28	–	+++*	+++*
7067_4_48	–	+	+++*
7067_4_21**	–	++*	+++*
7067_4_60**	++	+++*	+++*
SH46	–	++*	+++*
SH01	–	+++*	+++*
SH09	–	+	+++*
SH04	–	+++*	+++*
SH10	+	+++*	+++*
SH47	+	+++*	+++*
SH14**	–	++*	+++*
SH20**	+	++*	+++*
7067_4_39**	–	+	+++*

*Mutants observed after 24 h. 5 μl of each bacteriocin at 1 mg/ml was used in the spot-on-lown assay. Average Inhibition score (n = 3) indicates: “−” = No inhibition; “+” = Unclear zone; “++” = zone < 1 cm, and “+++” = zone ≥ 1 cm.

**Strains where H1 resistant mutants where isolated from 1 mg/ml spot-on-lawn inhibition zone.

### RseP is required for H1 antimicrobial activity

Previous studies have shown that the enterocins K1 and EJ97 require the membrane-bound protease RseP to recognize and kill enterococcal cells^23^. It is logical to assume that H1, a hybrid of K1 and EJ97, targets the same receptor. To test this, we heterologously expressed the *S. haemolyticus* RseP in *Lactobacillus plantarum* WCFS1^36^, which is resistant to the enterocin H1. As expected, *L. plantarum* expressing the *S. haemolyticus rseP*, showed sensitivity comparable to the parental *S. haemolyticus* strain, while *L. plantarum* harbouring the empty vector (pEV) was resistant to H1 (Fig. S1; Table 2).

**Table 2:** Minimal inhibitory concentration (MIC) values (µg/ml) of H1 towards different *L. plantarum strains*.

Strain	Relevant characteristics	H1 (µg/ml)
*L. plantarum* WCFS1	WT of host strain	> 50
*L. plantarum WCFS1/pEV*	Expressig empty vector	> 50
*L. plantarum* WCFS1/pSIP401_SHRseP	Expressing RseP from *S. haemolyticus* 7067_4_21	0.39

### The *rseP* gene is not the hot spot for mutations in the H1-resistant mutants

Although the role of RseP in the sensitivity of *S. haemolyticus* towards H1 was evident, we sought to confirm whether mutations were within *rseP* among the H1-resistant mutants. The *rseP* gene was therefore sequenced in a pool of 14 randomly selected H1-resistant mutants of *S. haemolyticus*. Surprisingly, only two mutants (4070 10_2 and 4071 1_4) had mutations within *rseP*, both with amino acid substitutions. In contrast, no mutation within *rseP* was found for the remaining H1-resistant mutants.To solve this puzzle, we performed whole-genome sequencing (WGS) on five additional H1-resistant mutants and the respective wildtype (WT) counterparts (Table 3). Surprisingly, four sequenced mutants contained mutations within the genes *ecsAB,* encoding subunits of the ATP-binding cassette (ABC) transporter Ecs^37,38^. The Ecs ABC transporter is known to be tightly connected with RseP in a secretory pathway in aerobic and facultative anaerobic Firmicutes, including staphylococci^39,40^. The last mutant contained a frame-shift mutation within *rseP* resulting in a truncated product (Table 3).

**Table 3:** Whole genome sequencing of selected H1 resistant mutants.

H1 resistant *S. haemolyticus* isolate	Mutations*	
	*rseP*	*escAB*
7076_4_21 M1	–	*escB*: c.855delT p.Phe285fs
7076_4_21 M2	–	*escB*: c.855delT p.Phe285fs
4069 M3	–	*ecsA*: c.113G>A p.Gly38Asp
4071 M3	c.1136_1139dupGTGG p.R381fs	–
4072 M3	–	*ecsB*: c.51_52insT p.Lys18fs

*: Intact gene, c: Coding DNA, p: Protein, del: deletion, fs: frameshift, >: Substitution, ins: Insertion, dup: duplication.

### Micrococcin P1 and garvicin KS potentiate the activity of H1

**Figure 2:**
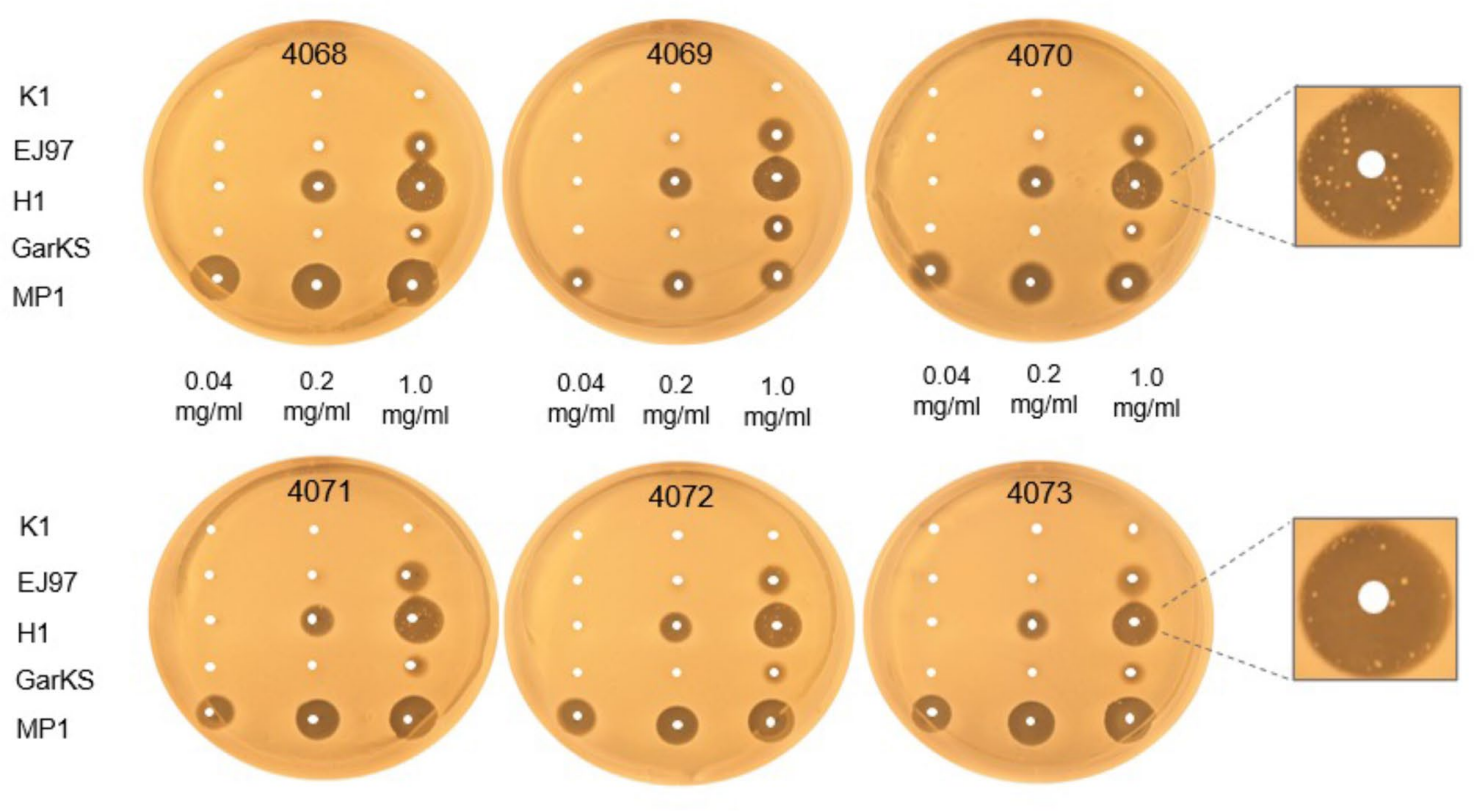
Comparison of the antimicrobial activities of the bacteriocins against *S. haemolyticus*. The antimicrobial activity of the indicated bacteriocins was assessed by “spot-on-lawn” assay against six *S. haemolyticus* strains, 4068–4073*.* The bacteriocins were applied at three different concentrations (0.04, 0.2, and 1.0 mg/ml), and 5 µl of each was spotted on the lawns of the *S. haemolyticus* strains. All strains developed resistance to H1 after 24 h incubation, as illustrated for strains 4070 and 4073.

The frequent resistance to H1 observed above, prompted us to investigate its synergistic effects in combination with other bacteriocins. To this end, the bacteriocins micrococcin P1 (MP1) and garvicin KS (GarKS) were chosen as they have been used in synergy studies before^20,41^. MP1 is a thiopeptide inhibiting protein synthesis^42–45^, while GarKS is a three-peptide bacteriocin with a yet unknown mode of action^46^. We initially evaluated the antimicrobial activity of the two bacteriocins against the six *S. haemolyticus* strains isolated from leprosy-associated plantar skin ulcers (4068–4073). For comparison, we also included the parental bacteriocins EJ97 and K1. We used the spot-on-lawn assay where these strains were challenged with three different concentrations of each antimicrobial (0.04, 0.2 and 1.0 mg/ml). As shown in Fig. 2, MP1 gave clear inhibition zones at all three concentrations while H1 gave inhibition only at the two highest concentrations. For GarKS and EJ97, some inhibition was seen only at the highest concentration. K1 did not result in any inhibition.

**Figure 3:**
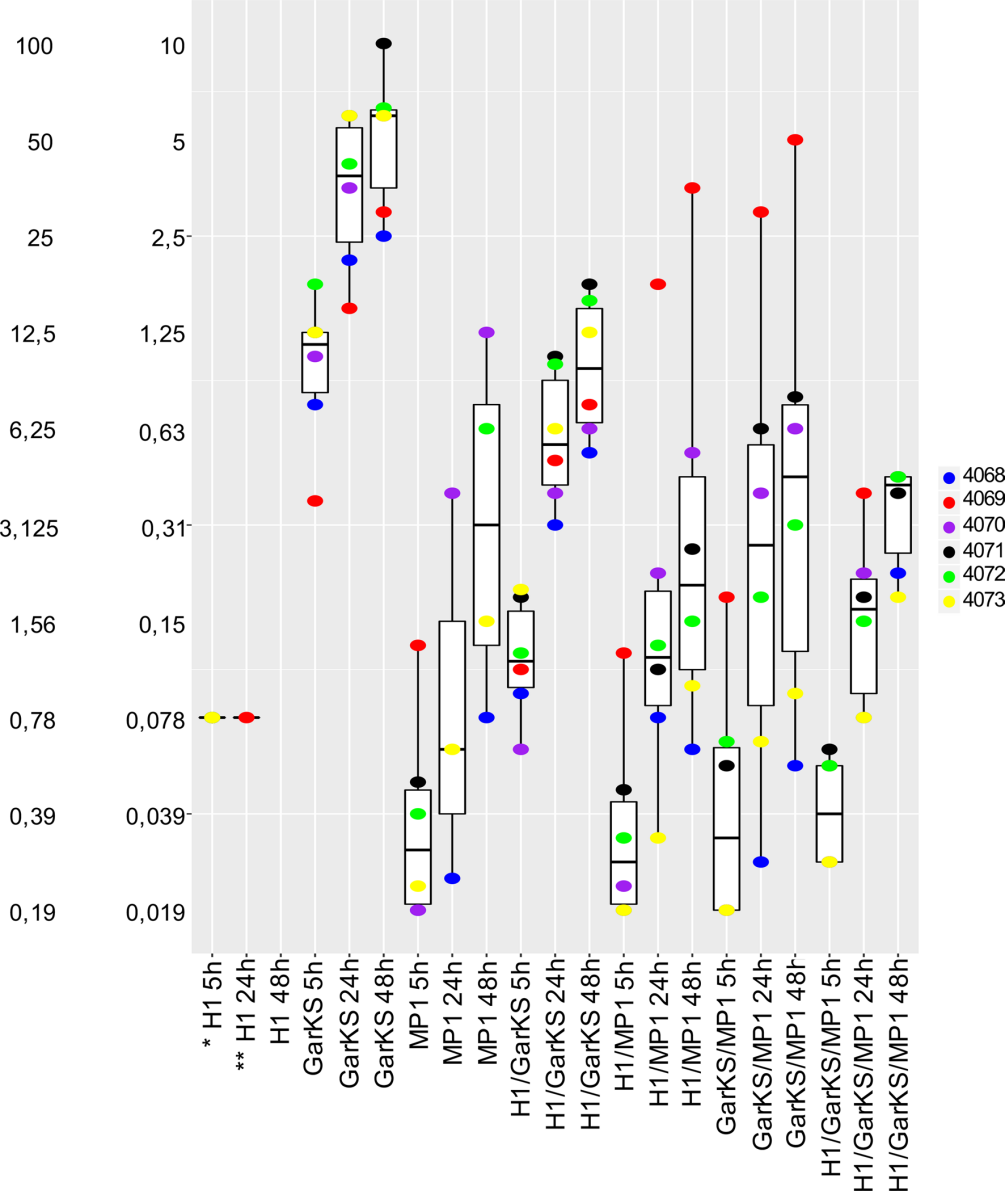
Boxplot of minimal inhibition concentrations (μg/ml) for the treatment with H1, garvicin KS, micrococcin P1 and their combinations against planktonic *S. haemolyticus*. The median distribution is indicated as thick line within boxes and the degree of variability (amplitude of the box or inter quartile region (IQR)) of the MIC_50_ values for the indicated strains obtained at three different time points: 5 h, 24 h and 48 h. Whiskers extending out the boxes mark the minimum and maximum observed values and the variability outside the middle 50% of values (whisker length). Outliers are represented as values that extend out of the whisker limit (1.5×IQR). Note that the treatment with the tricomponent combination (H1/GarKS/MP1) led to contained MIC values and inter-strain variability compared to the other treatments at all time-points. (*)Please note that for the treatment with H1 all strains were sensitive after a 5 h incubation (MIC_50_ = 0.78 μg/ml); (**) all strains were resistant after a 24 h incubation, except 4069 (MIC_50_ = 0.78 μg/ml); all strains were resistant after a 48 h incubation.

To investigate whether there was synergy between the three bacteriocins (H1, GarKS and MP1), a checkboard antimicrobial assay was performed. The selected strains were treated for 5, 24 and 48 h with a range of concentrations of H1, GarKS and MP1 alone or in different combinations. The results are summarized in Fig. 3 and further details on individual MIC_50_ values for the tested strains can be found in Table S3. After a 5 h incubation, MP1 showed the strongest antimicrobial activity, with MIC_50_ values ranging between 0.02 and 0.15 μg/ml, whereas H1 had a MIC_50_ of 0.78 μg/ml against all strains. Consistent with the antimicrobial test in Fig. 2, GarKS was the least active bacteriocin, with MIC_50_ values ranging between 3 and 24 µg/ml (Fig. 3; Table S3).

The prolonged exposure for 24 and 48 h resulted in a progressive increase in the MIC_50_ values for all treatments, a sign of resistance development. In agreement with the results above, H1 alone readily led to the generation of resistance among all tested strains (Fig. 3; Table S3). On the other hand, while the double combinatorial treatments (GarKS/MP1; H1/GarKS; H1/MP1) did not significantly increase the antimicrobial activity of the bacteriocins, combining all three bacteriocins (called the tricomponent combination) efficiently inhibited the microbial growth at all time-points. Interestingly, treatment with the tricomponent combination led to contained MIC_50_ values, with reduced inter-strain variability and displayed no sign of resistance development (Fig. 3). This was particularly evident at the 24 and 48 h time-points compared to the other treatments (Table S3).

Taken together these data indicate that the tricomponent combination had not only a superior antimicrobial activity but was also able to prevent resistance development.

### The tricomponent combination is effective against *S. haemolyticus* biofilms

The ability to form biofilms is a widespread virulence factor among CoNS; it is significantly associated with the colonization of prosthetic implants, increased antimicrobial resistance and therapeutic failure^47^. Among *S. haemolyticus* the genetic elements conferring adhesive properties and ultimately the ability to form biofilms are widely distributed^48,49^. In order to assess whether our clinical *S. haemolyticus* strains were indeed able to form biofilms in vitro, we performed a crystal violet assay on biofilm-associated cells^50^. Twelve *S. haemolyticus* isolates were included in the study, six of which were isolated from leprosy-associated skin ulcers in India (4068–4073), four were isolated from bacteremia patients and two were commensial isolates. We also included the methicillin-resistant *S. aureus* strain USA300 and a leprosy-associated *S. arlettae* strain as controls for a strong and a poor biofilm-former, respectively (Fig. S2)^41,51^. Using the crystal violet assay, the strong biofilm-former *S. aureus* USA300 produced an average OD value of over 3 (cut-off value for biofilm formers = 1), while the poor biofilm-former *S. arlettae* gave an average OD value of 0.5. All *S. haemolyticus* strains formed biofilms comparable to that of *S. aureus* USA300, except 7067_4_60 which produced an average OD value of 2 (Fig. S2). These results indicate that most pathogenic *S. haemolyticus* strains are strong biofilm-formers.

**Figure 4:**
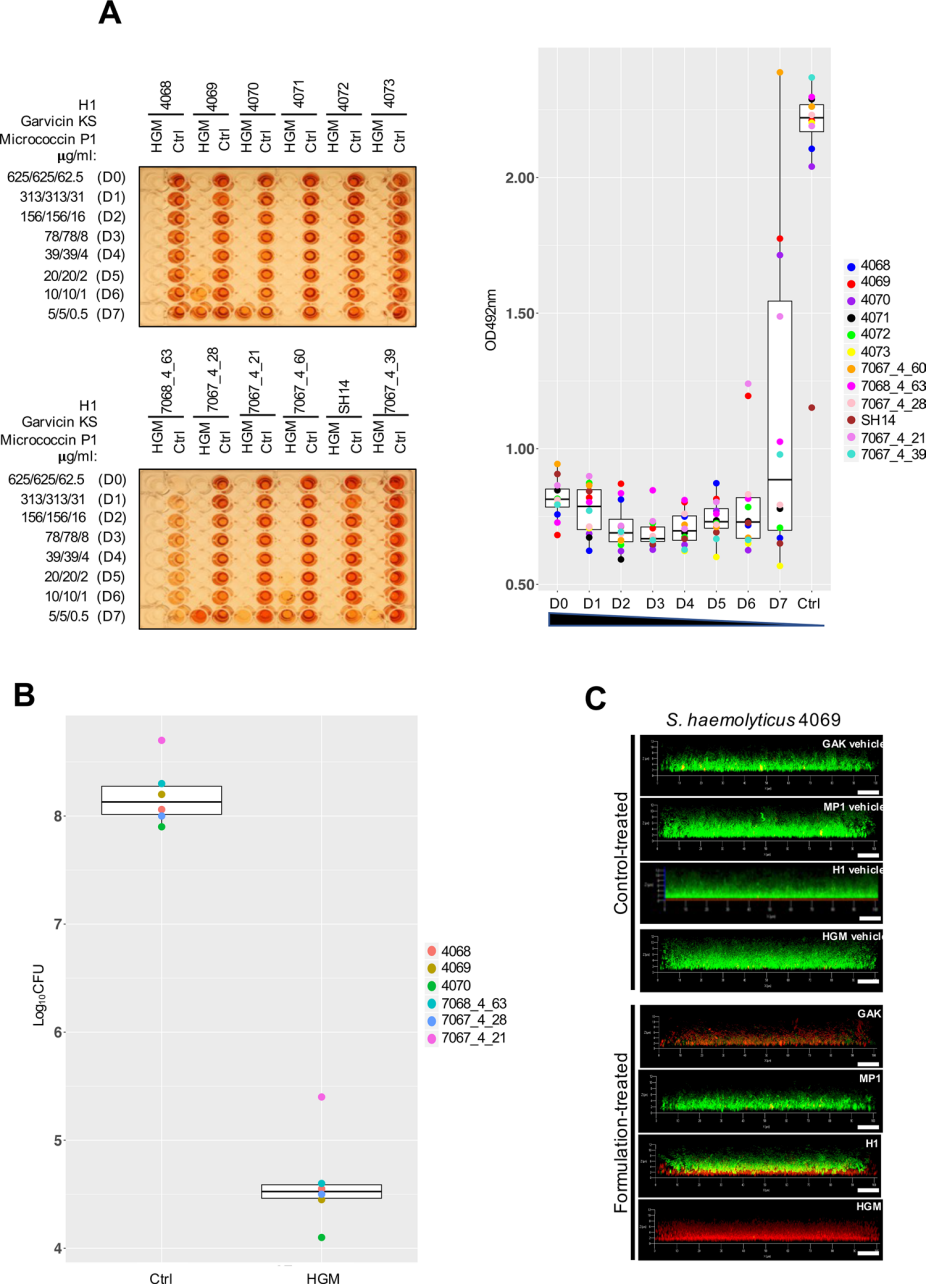
The tricomponent antimicrobial combination effectively inhibits the metabolic activity and viability of *S. haemolyticus* biofilm-associated cells in vitro. (**A**) The left panels show representative images of BOAT assays performed upon 24 h treatment with the tricomponent combination (HGM: H1, GarKS, MP1) for the indicated strains. The concentration (μg/ml) of the individual antimicrobials in the dilutions (dilution factors: D0 to D7) is indicated on the far left of the images. As controls, the assay was performed using the vehicles at their final concentrations (Ctrl). The development of red color indicates the retention of metabolic activity, and its quantification was performed by spectrophotometry at OD_492_. Metabolic activity values were plotted as a function of the dilution factor for the antimicrobial combination (right panel). Shown is the median distribution (thick line within boxes) and the degree of variability (amplitude of the box or inter quartile region (IQR)) of the metabolic activities for the indicated strains measured at increasing dilution factors (D0–D7). The boxplot components (whiskers and outliers) are displayed as specified in Fig. 3. Note that the treatment maintained the metabolic activitiy under detectable levels up to D6 for most strains. (**B**) The logarithmic colony formation unit (Log_10_CFU) was calculated after the BOAT assays were performed for the indicated strains. BOAT assays were performed as in (**A**), and the boxplot shows the median distribution of the Log_10_CFU values. The antimicrobial concentrations in the tricomponent combination were 625 μg/ml for H1 and garvicin KS and 62.5 μg/ml for micrococcin P1. The control was performed by using the antimicrobials vehicles at their final concentration. (**C**) The *S. haemolyticus* strain 4069 was allowed to form biofilms on glass-bottomed chambers for 24 h, prior to treatment with the indicated antimicrobials (lower set of panels) or the respective control-vehicles (upper set of panels) as detailed in (**B**). The biofilms were subsequently stained using the LIVE/DEAD biofilm staining kit and confocal microscope images were taken using a 63 × oil immersion objective. Scale bars correspond to 10 μm.

Given the strong antimicrobial effect of the tricomponent combination identified above, we wanted to examine its efficacy against biofilm-associated *S. haemolyticus* cells. We used a modified version of the biofilm-oriented antimicrobial test (BOAT)^52,53^ which allows the quantification of the metabolic activity (via the metabolic indicator triphenyl-tetrazolium chloride) within residual live cells within the biofilms after antimicrobial treatment. To do this, the *S. haemolyticus* biofilms were first allowed to form for 24 h, then the biofilms were treated with the antimicrobials for 5, 24 or 48 h, before the BOAT assay was performed (Fig. 4A; Fig. S3). We exposed biofilm-associated cells to a serial dilution of the tricomponent combination, starting with the highest concentrations (D0) being 625 μg/ml for H1 and garvicin KS and 62.5 μg/ml for micrococcin P1. These high values were 100 times higher the MIC_50_ values for planktonic cells, accounting for the higher resilience to antimicrobial treatment of biofilm-associated cells^15,41,54^. After 5 h the metabolic activity of all tested strains was very low or undetectable at all dilutions, except for 4069, which showed a residual metabolic activity at the highest dilution factors (D6 and D7) (Fig S3A). After a prolonged incubation for 24 and 48 h, most strains showed resilience but only at the lowest concentrations D5 and D7. For strains 4071–4073, 7068_4_63 and SH14, little or no metabolic activity was seen at all dilutions (Fig. 4A; Fig. S3B).

Bacterial cells can remain dormant (with very low or no metabolic activity) within the biofilms^55,56^. If this was the case in our biofilm settings, these cells would be overlooked by the BOAT assay. To examine this issue, biofilm-associated *S. haemolyticus* cells were treated with the tricomponent combination at concentration D0 (e.g. 625 μg/ml for H1 and garvicin KS and 62.5 μg/ml for micrococcin P1) for 24 h. This was followed by CFU counting to identify potential survinving cells in the treated biofilms. As shown in Fig. 4B, this treatment led to a dramatic and statistically significant (*p* < 0.0001) reduction of the biofilm-associated cell viability, with a drop in Log_10_CFU values ranging between 3.3 and 3.8 compared with the non-treated control for all six *S. haemolyticus* strains. These results were further confirmed by LIVE/DEAD biofilm staining followed by confocal microscopy analysis (Fig. 4C) which indeed showed only dead (red) cells within the biofilms when treated with the tricomponent combination. On the other hand, mixtures of live (green) and dead cells were observed when treated with the single antimcrobials. It is interesting to note that the treatment with H1 led to the killing of the bacterial cells in the deepest layers of the biofilm while preserving the cell viability in upper layers. This effect could not be observed for GarKS and MP1, possibly reflecting a mode of action and/or pattern of penetration within the biofilm specific for H1.

## Discussion

In this study we described the generation of a hybrid bacteriocin (H1) obtained by the fusion of the N- and C-terminal parts of the enterocins K1 and EJ97, respectively. The resulting H1 shows much better activity against the important human pathogen *S. haemolyticus* compared with EJ97 and especially K1 (Table 1). Both parental enterocins are class-II leaderless antimicrobial peptides. They share high sequence homology with each other and both use the membrane-bound RseP as a receptor on target cells^23^. We have previously shown that within the LsbB-group which contains LsbB, EJ97 and K1, the C-terminal part of these bacteriocins dictates their target specificity while the N-terminal part contains an amphiphilic alpha-helix which likely has the pore-forming function^23,57–60^. Since H1 was composed of the C-terminal half of EJ97 and the N-terminal half of K1, it is reasonable to think that the hybrid bacteriocin would have an inhibition spectrum similar to EJ97. This notion was generally in line with the result presented in Table S1 which showed an overlapping inhibition spectrum between H1 and EJ97. Nevertheless, there were some few important differences: H1 displayed an activity against certain strains of *B. cereus*, *C. pisciola*, *L. garvieae* and *S. arlettae* that overlapped with K1. On the other hand, compared to both parental enterocins, H1 showed a higher activity against members of the genera *Staphylococcus* (*S. aureus, S. epidermidis* and *S. haemolyticus*) and *Streptococcus* (*S. termophilus* and *S. uberis*) (Table S1). This indicates that the K1’s N-terminal half in H1, to some extent, also modulates the specificity of H1. Importantly, our analysis on 27 clinical *S. haemolyticus* strains indicated that the antimicrobial activity of K1 and EJ97 was highly strain-dependent, whereas H1 retained a high level of antimicrobial activity against all *S. haemolyticus* strains (Table 1).

It is worth mentioning that another hybrid peptide (H2) with an inverse order to H1 was also made, i.e., with an N-terminal half from EJ97 and a C-terminal half from K1. However, unlike H1, the inverted peptide showed in general very low activity (data not shown), and therefore no further analysis was carried on this version. At present, we do not know why H2 had low antimicrobial activity, but this is beyond the scope of the present study.

Despite the increased activity against *S. haemolyticus*, the antimicrobial potential of H1 was unfortunately hampered by the rapid development of resistance. Previous studies highlighted that multicomponent antimicrobial combinations offer a superior efficacy in terms of antimicrobial activity and prevention of resistance development^20,41,61,62^. In this study, we assessed the efficacy of H1 in combination with garvicin KS and micrococcin P1. The former is a multi-peptide bacteriocin composed of three non-modified peptides and belongs to the leaderless family of bacteriocins (class IId)^46^. Its mode of killing has not been elucidated in detail. However, it is supposed to disrupt the cell membrane integrity leading to the leakage of intracellular fluids, followed by cell lysis and death (unpublished data). On the other hand, micrococcin P1 belongs to a class of microbial ribosomally-synthesized and post-translationally modified peptides (RiPPs) known as thiopeptides^63^; a group of protein synthesis inhibitors^42–45^. The combination of H1 with garvicin KS and micrococcin P1 elicited a significant reduction of the antimicrobial concentrations required to effectively inhibit the growth of both planktonic and biofilm-associated *S. haemolyticus* cells.

Consistent with our recent study on *S. aureus* biofilms^41^, a discrepancy between the inhibition of biofilm-associated metabolic activity and cell viability could be observed. Indeed, treatments that led to a complete abolishment of metabolic activity did not translate directly into a corresponding annihilation of the cell viability quantified by CFU counting. A likely explanation for such an effect is biofilm-associated cell dormancy. Bacterial dormancy is a well-documented phenomenon, which significantly contributes to the long-term persistence of bacterial cells^55,56^. The dormant phenotype is characterized by low levels of metabolic activity, which confers the reduced susceptibility to antimicrobials as most antibiotics only attack active metabolic pathways such DNA, RNA, protein, cell wall synthesis^56,64^. Therefore, it is conceivable that if persister cells do exist within the biofilms in our model, their metabolic activity might not be tracked in the BOAT assays; however, metabolic reactivation will occur once cells are transferred to an appropriate growth-promoting environment^65,66^.

Bacteriocin-based antimicrobial combinations have been widely explored against a variety of pathogenic bacteria, including biofilm-forming strains^67,68^. However, these studies primarily focused on investigating the interaction between bacteriocins and different classes of antibiotics or other bioactive molecules^68–72^. Recently, also garvicin KS and micrococcin P1 have been tested in combination with penicillin G against MRSA, showing a potent antimicrobial activity^20,41^. To our knowledge, the antimicrobial combination described in the present work is the first bacteriocin-based and antibiotic-free example. In our view, this has a significant advantage as it complies with the health authorities’ general recommendations: namely to reduce or, where possible, avoid the use of antibiotics to treat bacterial infections. Similar recommendations were set by the European Commission (2015/C 299/04) also in veterinary medicine, particularly in the food production chain. In treating certain medical conditions, such as mastitis in dairy cows, antibiotic therapy will result in drug accumulation into the milk, leading to short-term withdrawal of milk from antibiotic-treated cows and as a consequence, significant economic loss for farmers. Thus, our bacteriocin-based and antibiotic-free approach can be a valuable solution in this context.

**Figure 5:**
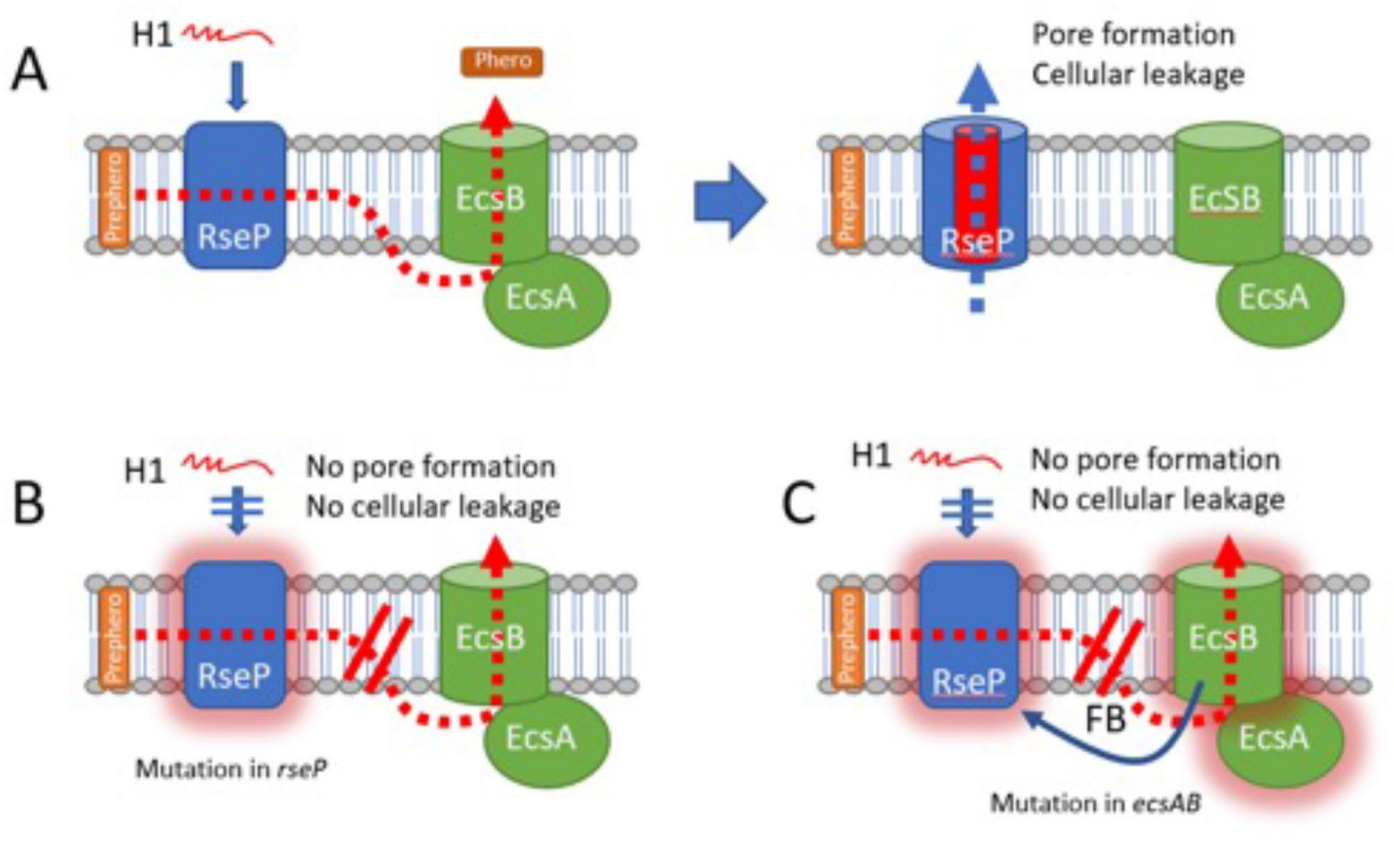
Proposed model for the EcsAB mediated H1-resistance in *S.haemolyticus*. (**A**) RseP is a inner-membrane protease working together the ABC-transporter EcsAB to cleave the hormone prepeptide (Prephero) and export the mature hormone peptide (Phero) to the external milieu. RseP is acting also as the receptor for the bacteriocin H1 which forms pores and causes lethal cellular leakage across the membrane. (**B**) When *rseP* is mutated resulting in a non-functional receptor (RseP with glow), cells become resistant to H1. (**C**) When the *ecs* system is mutated, the malfunctional ABC transporter can no longer export the hormone peptide. This jammed situation of hormone prepeptide/peptide within the membrane results in a feedback (FB) loop somehow causing RseP inactivity. An inactive RseP (RseP with glow) loses its function as a bacteriocin receptor, and cells therefore become resistant to H1.

Our complementation studies using *L. plantarum* confirmed that RseP was indeed the target for H1 on the *S. haemolyticus* cells and was essential for its antimicrobial activity. Therefore it was very surprising to reveal that most *S. haemolyticus* H1-resistant mutants did not possess a disruptive mutation within the *rseP* gene. By WGS, we found that 4 out of 5 mutants contained mutations in the *ecsAB* gene pair, which encodes components of the ATP-binding cassette (ABC) transporter Ecs. The fact that mutations accumulated within the ABC transporter abolished the sensitivity to H1 might indicate a molecular link between Ecs and RseP. Interestingly, similar observations have also been found in other studies. In *Bacillus subtilis* the perturbation of the *ecsAB* gene locus leads to the disruption of RasP function (RasP is a RseP homolog). In addition, inactivation of *rasP* led to an overlapping phenotype with *B. subtilis* strains carrying mutations in *ecsA*^39^. In *Enterococcus faecalis*, EcsAB and Eep (the enterococcal homolog of RseP) are functionally connected in a quorum sensing pathway involved in conjugation^73^. Eep is responsible for the processing of several pheromone precursors, including cCF10, cAD1 and cAD1^30,74^, and the secretion of the mature sex pheromones is then mediated by EcsAB^73^, resulting in the conjugative transfer of mobile genetic elements; including antibiotic resistance determinants ^30,75^. Similarly, the inactivation of an Eep-like metalloprotease in *Streptococcus gordonii* abolishes the secretion of the cAM373 pheromone^76^. Based on these similarities, it is tempting to speculate that a similar mechanism is involved in the H1-resistant *S. haemolyticus ecs* mutants. In such a model as depicted in Fig. 5A, EcsAB is working down-stream of RseP, to export an unknown product provided by RseP. Mutations in *rseP* will destroy its receptor function thereby allow cells becoming resistant to H1 (Fig. 5B). Mutations in *ecsAB* will entail the accumulation of RseP-processed peptides in the cell membrane that may affect, in a feedback maner, the functionality of RseP, ultimately rendering RseP no longer suitable as the bacteriocin receptor (Fig. 5C). This model can also probably be applied to enterococci, since *E. faecalis* mutants which are resistant to EJ97 contain mutations either within *ecsAB* or *rseP*; however, a much higher frequency was observed in the latter^77^. Therefore, it was unexpected to find high frequency of mutations in *ecs* relative to *rseP* in *S. haemolyticus*. The molecular nature behind the different mutation rates in *rseP* and *ecsAB* between these two pathogens is unknown, yet it might reflect the different roles of RseP in these two pathogens. Undoubtfully, the role of RseP and its connection with the Ecs system deserves further investigation in future studies.

The high degree of genome plasticity of *S. haemolyticus*^3^ allows this bacterium to readily acquire resistance mehcanisms toward many different antibiotics. Here we demonstrated that using multiple bacteriocins with different modes of action can overcome the resistance problem in *S. haemolyticus*. The tricomponent combination with the bacteriocins H1, MP1 and GarKS proves effective both against planktonic and biofilm-associated cells, the latter being much troublesome in therapeutic treatment due to the protective biofilm layers. Our data indicate that the antimicrobials in the mixture were able to peneterate the protective biofilm layer and kill the bacterial cells hiding underneath. Antimicrobial peptides are ubiquitous in nature, and they are very diverse in terms of amino acid sequence, structure, modification, and mode of action. By genetic means, or by de novo synthesis, new hybrid antimicrobial peptides can be easily made to improve potency and/or alter target specificity, as demonstrated in the present study with H1. This approach can be a precious path to create novel antimicrobials and combat bacterial infections and antibiotic resistance.

## Materials and methods

### Bacterial strains and cultivation conditions

Bacterial strains used in this study are listed in Tables S1, S3 and S4. The following strains were cultivated in Brain heart infusion (BHI) broth (Oxoid): all indicator strains (30 °C, with shaking), all clinical *S. haemolyticus* isolates (Table S2) and *Escherichia coli* (DH5α, TG1) (37 °C, with shaking). *Lactobacillus plantarum* (WCFS1) was cultivated in MRS broth (Oxoid) at 37 °C without shaking. Agar plates were prepared by supplementing the appropriate broth with 1.5% (w/v) agar (VWR chemicals). When appropriate, erythromycin was added to a final concentration of 200 μg/ml for *E.coli* and 10 μg/ml for *L. plantarum*.

### Bacteriocins and antimicrobial assays

Bacteriocins used in this study are listed in Table S5. K1^23^, EJ97^31^, H1 and garvicin KS peptides (Gak-A, -B and -C)^46^ were synthesized by Pepmic Co., Ltd, China, with > 95% purity. These bacteriocins were all solubilized in 0.1% (vol/vol) trifluoracetic acid (TFA; Sigma-Aldrich). Micrococcin P1^78^ was purchased from Cayman Chemical, Michigan, USA with^3^ 95% purity and solubilized in a 50% (v/v) mixture of isopropanol (Merck) with 0.1% (v/v) TFA (Sigma-Aldrich) at a stock concentration of 1 mg/ml.

A semi-quantitative spot-on-lawn inhibitory assay was performed on 51 bacterial indicator strains (Table S1) to determine the bacteriocins inhibition spectra. Overnight cultures were diluted 1:100 in BHI soft agar and distributed on BHI agar plates. Once solidified, bacteriocins with various concentrations were applied on designated spots. The plates were incubated at appropriate temperature O/N and inhibition zones were measured the following day. To determine minimal inhibitory concentration (MIC_50_) of H1 against clinical *S. haemolyticus* isolates (Table S2), as well as strains heterologously expressing RseP (Table S4), the microtiterplate antimicrobial assay was performed as previously described^41,79^. MIC_50_ was defined as the minimal bacteriocin concentration needs to inhibit the growth of a bacterial strain by at least 50%.

Synergistic interactions between antimicrobials were determined using the fractional inhibition concentration (FIC) as previously described^80^. The FIC values were determined using a mirotiterplate checkerboard assay. Briefly, antimirobial A was applied in wells A1–H1 then diluted two-fold in wells 2–12. In a second microtiter plate, antimicrobial B was applied in wells A1–A12, then diluted two folds in wells B–H. Fifty μl of the serial diluted antimirobial A was transferred to the wells of a third microtiter plate, except for wells H1–H12. Similarly, 50 μl of the serial diluted antimicrobial B was transferred to the same microtiter plate, except for wells A1–A12. Wells H1-12 and wells A1-12 contained only one of the antimicrobials and was used to estimate the MIC value of the pure substance. After combining the antimicrobials, 100 μl of a 1:25 diluted O/N of *S. haemolyticus* was transferred to each well of the third microplate, while 50 μl of medium was added to wells H1-12 and A1-12. The plates where incubated at 37 °C for 5, 24 or 48 h before the MIC values were scored. FIC values were calculated as follows: FIC = FICa + FICb + FICc, where the FICa means MIC of A in combination/MIC of A alone, FICb means MIC of B in combination/MIC of B, and FICc means MIC of C in combination/MIC of C alone. Effects were considered as synergistic if FIC was ≤ 0.5 for two components mixture^81^ and ≤ 0.75 for three components mixture^82^.

### Isolation of H1-resistant mutants

During the spot-on-lawn inhibitory assay on plates, H1-resistant *S. haemolyticus* isolates were observed within the inhibition zones. Resistant colonies within the inhibition zones with various H1-concentrations (1 mg/ml or 10 mg/ml) were re-streaked to obtain pure colonies. Glycerol stocks (15% glycerol v/v) of the pure cultures were made and stored at − 80 °C until use.

### DNA sequencing

For *rseP* sequencing, *rseP* was PCR amplified using pure colonies or gDNA isolated from H1-resistant *S. haemolyticus* strains as a template. gDNA was isolated from the resistant mutants using NucleoSpin Genomic DNA kit from Microorganisms (Macherey–Nagel). *rseP* was amplified using Q5 Hot Start High-Fidelity DNA polymerase (New England Biolabs). PCR amplicons were purified using NucleoSpin Gel and PCR clean-up kit (Macherery-Nagel), sequenced at Eurofins GATC, Germany, and analysed using CLC Main Workbench (Qiagen) (https://digitalinsights.qiagen.com). Primers used for sequencing and PCR amplification are listed in Table S6. For whole genome sequencing, genomic DNA was sequenced using Illumina Next Generation Sequencing. Contigs were assembled using Unipro UGENE^83^, while SnapGene software (GSL Biotech), Blast (NCBI) and UniProt was applied in further downstream analysis.

### Heterologous expression of *rseP*

*rseP* (Uniprot: Q4L5W4) derived from *S. haemolyticus* 7067_4_21 was heterologously expressed in the naturally H1-resistant *L. plantarum* WCFS1^36^ using the vector pSIP expression system^84,85^. First, pLp1261_InvS, a pSIP401 derivative^84,86^, was linearized using FastDigest Restrictiction Enzymes NdeI and Acc65I (ThermoFisher Scientific) to isolate the vector part. Second, *rseP* was amplified by PCR using specially designed In-Fusion Primes (Table S6), giving amplicons with complementary ends to the linearized vector. The In-Fusion HD cloning Kit (Takara Bio) was used to fuse *rseP* into the linearized vector, yielding pSIP401_SHRseP. The plasmid was subsequently transformed to *E.coli* TOP10 (ThermoFischer Scientific). Plasmid DNA from *E. coli* was isolated using NucleoSpin Plasmid Kit (Macherey–Nagel). The DNA sequence of all PCR amplified fragments was verified by sequencing at Eurofins GATC, Germany, and before being electroporated into *L. plantarum* WCSF1 following the procedure described by Aukrust and Blom^87^.

### Biofilm formation assay

A microtiter dish biofilm formation assay was performed to determine the biofilm forming ability of *S. haemolyticus* strains. Briefly, the optical density measured at 600 nm (OD_600_) of O/N cultures was adjusted to 0.5 by diluting the cultures in 3% Tryptic Soy Broth (TSB; Sigma-Aldrich). This was followed by further diluting 10 ml of the relevant bacterial supentions in 90 ml of 3% TSB supplemented with 1% glucose in the appropriate wells of a 96-well plate (Sarstedt) and incubated at 37 °C for 24 h. Following incubation, the O/N culture medium was removed and the presence of biofilms within the inoculated wells was first assessed visually. For quantification, the biofilms were washed twice with 100 μl of 0.9% NaCl, incubated at 55 °C for 1 h to fix the biofilm forming cells to the plastic surface and then stained with a 0.5% crystal violet solution for 10 min at room temperature. Excess crystal violet solution was removed, and the biofilms were further washed with 100 μl of 0.9% NaCl three times. The residual biofilm-bound crystal violet was then extracted by incubating the wells with 100 ml of absolute ethanol (Sigma-Aldrich) for 10 min. The extraction procedure was repeated twice, and the amount of crystal violet extracted was quantified at OD_600_. The quantification of the crystal violet released from the biofilm is a surrogate measure of the biofilm formation ability of a strain.

### Biofilm-oriented antimicrobial test (BOAT)

To assess the antimicrobial effects of bacteriocins on biofilm-associated *S. haemolyticus* cells, the metabolic activity of biofilm-associated cells was determined by using the biofilm-oriented antimicrobial test (BOAT), as previously described^41,52^. H1, garvicin KS and micrococin P1 were assessed individually or in combinations, using a serial two-fold dilution scheme of their concentration. Unless otherwise stated, the starting dilutions were 625 μg/ml for H1 and garvicin KS, 62.5 μg/ml for micrococcin P1. Biofilms were allowed to form for 24 h as described above and subsequently challenged with the bacteriocins, individually or in combinations, for 5, 24 or 48 h at 37 °C. As negative control, the assay was performed using the vehicles to their working concentration (i.e., without bacteriocins).

### Determination of persister cells within biofilms after BOAT

The procedure for the BOAT assay was repeated as above, with one exception. Instead of monitoring the residual metabolic activity of biofilm-associated cells after the bacteriocin challenge, bacteriocin-treated cells were resuspended in TSB and further serially diluted. The serial diluted bacterial cells were plated on BHI agar plates and incubated at 37 °C for 24 h. Colony forming units (CFU/ml) were used to quantify the remaining surviving cells after antimicrobial challenge.

### Confocal microscopy of biofilms

Biofilms were allowed to grow for 24 h as described above except that they were formed in the wells of chambered cover-glass plates (Thermo Fisher Scientific) before being challenged for 24 h with the single bacteriocins or combinations diluted in TSB. The biofilms were subsequently analyzed using the LIVE/DEAD Biofilm Viability Kit (Molecular Probes, Thermo Fisher Scientific) according to the manufacturer´s instruction. Z-stacks of the stained biofilms were then taken on a confocal laser scanning microscope (Zeiss), using a 488 nm argon laser line for exciting the SYTO-9 (green—live cells) dye and a 561 nm laser line for the propidium iodide (red—dead cells). The bacteriocin concentrations used were the same as described for the BOAT assay. The control vehicles for the single bacteriocins and their combination were used at the following concentrations: 0,0063% (v/v) TFA for H1; garvicin KS, 0,003% (v/v) TFA; 3,1% (v/v) 2-propanol for micrococcin P1.

## Supplementary Information

Supplementary Informations.
