# Supplementary material for: A bacteriocin-based treatment option for Staphylococcus haemolyticus biofilms
Source: Sci Rep. 2021 Jul 6;11:13909. doi: 10.1038/s41598-021-93158-z (PMC8260761; doi:10.1038/s41598-021-93158-z)
Supplement: Supplementary file 1 — Supplementary Informations. [file 41598_2021_93158_MOESM1_ESM.docx]

**
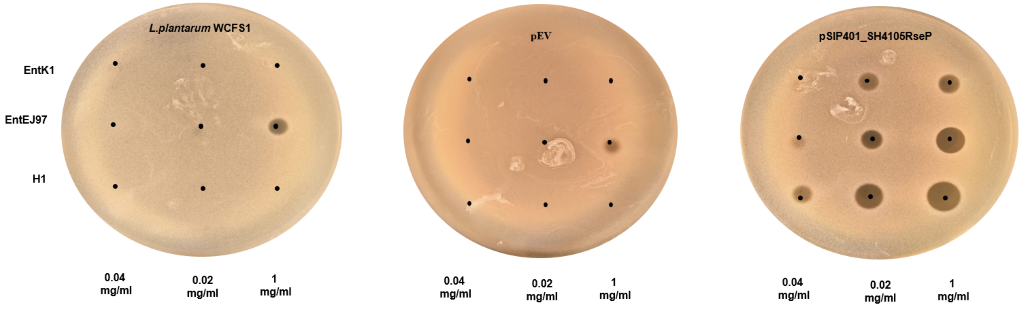
**

Figure S1. Heterologous expression of S. haemolyticus RseP in L. plantarum WCFS1. Spot-on-lawn assay on the wild-type L. plantarum WCFS1 (A), WCSF1 expressing the empty expression vector pEV (B) or pSIP401-SH4106RseP (a derivative of pEV) containing the S. haemolyticus rseP (C). rsep was derived from S. haemolyticus LMGT 4106. The antimicrobials were EJ97, K1 and H1, each at three concentrations (0.04, 0.2 and 1.0 mg/ml). Five ul of each concentration was applied to each spot. Inhibition is seen as dark zones around the white dots.

LMGT4068

LMGT4069

LMGT4070

LMGT4071

LMGT4072

LMGT4073

7068_4_63

7067_4_28

7067_4_21

7067_4_60

SH14

7067_4_39

USA300

LMGT4059

0

1

2

3

4

OD600nm

Figure S2. Evaluation of *S. haemolyticus* biofilm formation abilitiy. The indicated *S. haemolyticus* strains were allowed to form for 24 hours prior to staining with a crystal violet solution. The amount of dye bound to the cells is an indirect measure of the biofilm-forming ability and was quantified by optical density readings at 600 nm (OD_600_) for each strain. The bar chart shows the average values (± s.d.) obtained from three independent experiments. *S. aureus* USA300 and *S.arlettae* (4059) served as a positive and negative control for biofilm formation, respectively. Cut-off OD values for biofilm formation were set to 1.

Figure S3. Evaluation of the three-component combination antibiofilm activity. Representative images of BOAT assays (left panels) obtained after a 5 hour treatment (A) or after a 48 hours treatment (B) with the tricomponent antimicrobial combination (HGM - short for H1, GarKS and MP1) or with the control (Ctrl) vehicle. The assays were performed as described in Figure 4A. The boxplot in the right panel describes the recovery of metabolic activity after the antimicrobial treatment (refer to figure 4A for details). Note that the metabolic activity after a 5 hours treatemtn with the antimicrobials remained below detectable levels up to D6 for most stains. Increased levels of metabolic activity were seen when the treatment was extended to 48 hours, especially at high dilution factors (D5-D7).

**Table S1.** **Inhibition spectrum of K1, EJ97 and H1 as assessed by the spot-on-lawn assay against a collection of indicator species (n=50).**

| **Indicator species** | **Source or reference (*)** | **Bacteriocins (**)** | | |
| --- | --- | --- | --- | --- |
|  |  | **K1** | **EJ97** | **H1** |
| *Bacillus cereus* LMG 2805 | LMG | + | - | + |
| *B. cereus* LMG 2711 | LMG | + | - | + |
| *B. cereus* LMG 2731 | LMG | + | - | - |
| *Carnobacterium divergens* NCDO 2306 | NCDO | - | - | - |
| *C. piscicola* LMG 2332 | LMG | + | - | + |
| *Enterococcus avium* LMG 3465 | LMG | + | +++ | +++ |
| *E. faecalis* V583 | ^1^ | + | + | ++ |
| *E. faecalis* LMG 2333 | LMG | + | +++ | +++ |
| *E. faecalis* LMG 3331 | LMG | + | +++ | +++ |
| *E. faecalis* LMG 3330 | LMG | + | +++ | +++ |
| *E. faecalis* LMG 3332 | LMG | + | +++ | ++ |
| *E. faecium* L50 | ^2^ | ++ | +++ | ++ |
| *E. faecium* P13 | ^3^ | +++ | +++ | +++ |
| *E. faecium* P21 | ^4^ | ++ | +++ | +++ |
| *E. faecium* AL41 | ^5^ | ++ | +++ | +++ |
| *Esherichia coli* DH5α | ThermoFischer Scientific | - | - | - |
| *E. coli* TG1 | LMG | - | - | - |
| *Lactobacillus curvatus* LTH 1174 | ^6^ | +++ | +++ | ++ |
| *L. curvatus* LMG 2353 | LMG | +++ | +++ | ++ |
| *L. plantarum* LMG 2352 | LMG | + | ++ | - |
| *L. sakei* LMG 2380 | LMG | ++ | - | - |
| *L. sakei* 148 | ^7^ | +++ | - | - |
| *L. salivarius* UCC118 | ^8^ | - | - | - |
| *Lactococcus garvieae* DCC43 | ^9^ | +++ | + | +++ |
| *L. lactis* LMG 2081 | LMG | - | - | - |
| *L. lactis IL1403* | ^10^ | +++ | +++ | +++ |
| *L. gelidum* LMG 2386 | LMG | - | - | - |
| *L. innocua* LMG 2785 | LMG | +++ | - | +++ |
| *L. innocua* LMG 2710 | LMG | ++ | +++ | +++ |
| *L. ivanovii* Li4 | Nofima | + | + | + |
| *L. monocytogenes* 400 | VET | - | - | - |
| *L. monocytogenes* 223 | VET | - | - | + |
| *L. monocytogenes* 279 | VET | - | - | + |
| *L. monocytogenes* EDGe | ^11^ | + | +++ | +++ |
| *L. monocytogenes* 403 | VET | - | - | + |
| *Staphylococcus arlettae* LMG 4134 | LMG | - | + | - |
| *S. epidermidis* LMG 3522 | LMG | - | + | ++ |
| *S. haemolyticus* LMG 4133 | LMG | + | ++ | +++ |
| *S. homonis* LMG 3129 | LMG | ++ | +++ | +++ |
| *S. simulans* LMG 3233 | LMG | - | - | - |
| *S. aureus*  LMG 3326 | LMG | - | + | ++ |
| *S. aureus* LMG 3328 | LMG | - | - | + |
| *S. aureus* LMG 3263 | LMG | - | + | + |
| *S. aureus*  LMG 3325 | LMG | - | - | - |
| *S. aureus*  LMG 3329 | LMG | - | - | + |
| *S. aureus*  ATCC14458 | Nofima | - | - | - |
| *Streptococcus dysgalactiae* 7649-3 | MLM | - | - | - |
| *S. thermophilus* Sfi13 | ^12^ | - | - | +++ |
| *S. uberis* 8008-1 | MLM | - | - | ++ |
| *S. uberis* 7904-3 | MLM | + | - | ++ |
| *LMG: Laboratory of Microbial Gene Technology, Norwegian University of Life Sciences  NCDO: National Collection of Dairy Organisms, United Kingdom  Nofima: Norwegian Food Research Institute, Norway  VET: Faculty of Veterinary Medicine, Norwegian University of Life Science  MLM: Mastitis laboratorium in Molde, Norway  ** 5 µl of each bacteriocin at 1 mg/ml. Average inhibition score (n=3) indicates: “-“  = No inhibition; “+” = Unclear zone;  “++” = zone < 1 cm;  “+++”= zone > 1 cm. | | | | |

##

**Table S2. Minimal inhibitory concentration (MIC) values (µg/ml) of H1 towards clinical and commensial *S. haemolyticus* isolates***.*

| ***S.haemolyticus* strain** | **Isolation source (*)** | **MIC_50_   H1 ( μg/ml)** | **ENA**  **Acc. No.** | **Reference** |
| --- | --- | --- | --- | --- |
| 7067_4_39 | Blood culture, OUH | 0.78 | ERS066281 | ^(13)^ |
| SH20 | Commensal, skin, UNN | 0.78 | GCA_903969795 | ^(13)^ |
| SH14 | Commensal, skin, UNN | 0.78 | GCA_903969855 | ^(13)^ |
| SH47 | Commensal, skin, UNN | 0.10 | GCA_903969995 | ^(13)^ |
| SH10 | Commensal, skin, UNN | 0.10 | GCA_903969755 | ^(13)^ |
| SH04 | Commensal, skin, UNN | 0.78 | GCA_903969785 | ^(13)^ |
| SH09 | Commensal, skin, UNN | 0.78 | GCA_903969725 | ^(13)^ |
| SH01 | Commensal, skin, UNN | 0.78 | GCA_903969685 | ^(13)^ |
| SH46 | Commensal, skin, UNN | 0.78 | GCA_903969985.1 | ^(13)^ |
| 7067_4_60 | Blood, Switzerland | 0.10 | ERS066392 | ^(14)^ |
| 7067_4_21 | Commensal, Japan | 0.39 | ERS066353 | ^(14)^ |
| 7067_4_48 | Blood, OUH | 0.78 | ERS066290 | ^(14)^ |
| 7067_4_28 | Blood, OUH | 0.78 | ERS066270 | ^(14)^ |
| 7067_4_71 | Urine, OUH | 0.78 | ERS066313 | ^(14)^ |
| 7076_4_67 | Blood, UNN | 0.78 | ERS066309 | ^(14)^ |
| 7067_4_84 | Blood, United Kingdom | 0.78 | N/A | ^(14)^ |
| 7067_4_49 | Blood, OUH | 0.78 | ERS066291 | ^(14)^ |
| 7067_4_66 | Blood, UNN | 0.78 | ERS066308 | ^(14)^ |
| 7067_4_63 | Blood, Switzerland | 0.78 | ERS066395 | ^(14)^ |
| 7067_4_56 | Blood, OUH | 0.78 | ERS066298 | ^(14)^ |
| 7068_7_48 | Blood, Switzerland | 0.78 | ERS066380 | ^(14)^ |
| 4068 | Leprosy-associated plantar skin ulcers, BPHRC | 0.39 | N/A | This study |
| 4069 | Leprosy-associated plantar skin ulcers, BPHRC | 0.78 | N/A | This study |
| 4070 | Leprosy-associated plantar skin ulcers, BPHRC | 0.78 | N/A | This study |
| 4071 | Leprosy-associated plantar skin ulcers, BPHRC | 0.78 | N/A | This study |
| 4072 | Leprosy-associated plantar skin ulcers, BPHRC | 0.78 | N/A | This study |
| 4073 | Leprosy-associated plantar skin ulcers, BPHRC | 0.78 | N/A | This study |
| * OUH: Oslo University Hospital, Rikshospitalet, Norway; UNN: University hospital of North Norway; BPHRC: Blue Peter Public Health and Research Centre, India | | | | |

**Table S3.** **MIC values (μg/ml) determined for planktonic cells after 5, 24 or 48 hour exposure to H1, GarKS, MP1 or the** **indicated combinations.**

| **Antimicrobial** | **Strains** | | | | | | | | | | | | | |  |  |
| --- | --- | --- | --- | --- | --- | --- | --- | --- | --- | --- | --- | --- | --- | --- | --- | --- |
|  | 4068 | 4069 | 4070 | | | 4071 | | | 4072 | | | 4073 | | |  |  |
| **Individual component** |  |  |  | | |  | | |  | | |  | | |  |  |
| H1 5h  24h  48h | 0.78  >100  >100 | 0.78  0.78  >100 | | 0.78  >100  >100 | | | 0.78  >100  >100 | | | 0.78  >100  >100 | | | 0.78  >100  >100 | |  |  |
| GarKS 5h  24h  48h | 6.5  24  25 | 3.3  13  26 | 12  48  51 | | | 12.5  51  100 | | 23.6  49  52 | | | | 12.5  51  51 | | | | |
| MP1 5h  24h  48h | 0.02  0.023  0.078 | 0.15  >10  >10 | 0.02  0.33  0.13 | | | 0.043  >10  >10 | | 0.039  >10  0.63 | | | | 0.022  0.072  0.14 | | | |  |
|  |  |  |  |  |  |  |  |  |  |  |  |  |  |  |  |  |
| **Combination** |  |  |  | | |  | | |  | | |  | | |  |  |
| H1 5h  24h  48h | 0.83  3.1  6 | 1.4  6  6.5 | 0.72  3.3  6.3 | | 1.6  12  24 | | | 1.5  12  13 | | | 1.7  6.3  12 | | |  |  |  |
| GarKS 5h  24h  48h | 0.83  3.1  6 | 1.4  6  6.5 | 0.72  3.3  6.3 | | | 1.6  12  24 | | | 1.5  12  13 | | | 1.7  6.3  12 | | |  |  |
| FIC* 5h  24h  48h | 1.2  0.16  0.3 | 2.2  8.16  0.3 | 1.0  0.1  0.2 | | | 2.2  0.36  4.8 | | | 2.0  0.36  1.6 | | | 2.3  0.19  1.4 | | |  |  |
| **Combination** |  |  |  | | |  | | |  | | |  | | |  |  |
| H1 5h  24h  48h | 0.2  0.78  0.72 | 1.5  23  48 | 0.2  2.8  6 | | | 0.42  1.4  3 | | | 0.36  1.5  1.6 | | | 0.2  0.36  0.85 | | |  |  |
| MP1 5h  24h  48h | 0.02  0.078  0.072 | 0.15  2.3  4.8 | 0.02  0.28  0.6 | | | 0.042  0.14  0.3 | | | 0.036  0.15  0.16 | | | 0.02  0.036  0.01 | | |  |  |
| FIC* 5h  24h  48h | 0.36  0.35  0.1 | 2.0  29.7  5.3 | 0.36  0.11  0.1 | | | 0.64  0.028  0.06 | | | 0.55  0.03  0.04 | | | 0.36  0.05  0.01 | | |  |  |
| **Combination** |  |  |  | | |  | | |  | | |  | | |  |  |
| GarKS 5h  24h  48h | 0.2  0.32  0.68 | 1.6  25  50 | 0.2  3.3  6.3 | | | 0.68  6.3  6.5 | | | 0.73  1.6  3.1 | | | 0.2  0.73  0.83 | | |  |  |
| MP1 5h  24h  48h | 0.02  0.032  0.068 | 0.16  2.5  5 | 0.02  0.33  0.63 | | | 0.068  0.63  0.65 | | | 0.073  0.16  0.31 | | | 0.02  0.073  0.083 | | |  |  |
| FIC* 5h  24h  48h | 0.13  0.15  0.12 | 0.6  2.2  1.3 | 0.12  0.17  0.2 | | | 0.06  0.19  0.13 | | | 0.2  0.05  0.1 | | | 0.1  0.12  0.7 | | |  |  |
| **Combination** |  |  |  | | |  | | |  | | |  | | |  |  |
| H1 5h  24h  48h | 0.32  0.78  2.8 | 0.68  3.3  5.8 | 0.32  2.8  5.8 | | | 0.72  1.6  3.3 | | | 0.68  1.6  5.8 | | | 0.33  0.78  1.6 | | |  |  |
| GarKS 5h  24h  48h | 0.32  0.78  2.8 | 0.68  3.3  5.8 | 0.32  2.8  5.8 | | | 0.72  1.6  3.3 | | | 0.68  1.6  5.8 | | | 0.33  0.78  1.6 | | |  |  |
| MP1 5h  24h  48h | 0.032  0.078  0.28 | 0.068  0.33  0.58 | 0.032  0.28  0.58 | | | 0.072  0.16  0.33 | | | 0.068  0.16  0.58 | | | 0.033  0.078  0.16 | | |  |  |
| FIC** 5h  24h  48h | 0.62  0.38  0.5 | 1.12  4.5  0.4 | 0.6  0.17  0.2 | | | 1.2  0.06  0.1 | | | 1.1  0.06  0.26 | | | 0.6  0.14  0.15 | | |  |  |
| * Synergy with fractional inhibition concentration (FIC) ≤ 0.5. ** Synergy with fractional inhibition concentration (FIC) ≤ 0.75. | | | | | | | | | | | | | | |  |  |

**Table S4. Bacterial strains and plasmids used for heterologous expression of RseP.**

| **Strain or plasmid** | **Relevant characteristics (*)** | **Reference** |
| --- | --- | --- |
| Strain |  |  |
| *S.haemolyticus* hi7076_4_21 | Template for *rseP* | ^14^ |
| *E. coli* TOP10 | Cloning host | ThermoFischer Scientific |
| *L. plantarum* WCFS1 | Host strain | ^15^ |
| Plasmid |  |  |
| pEV | Em^r^; empty expression vector, a derivative of pSIP401 | ^16^ |
| pLp1261_InvS | Em^r^; a derivative of the *spp*-based expression vector pSIP401. | ^16,17^ |
| pSIP401_SHRseP | Em^r^; a derivative of pLp1261_InvS, containing a *S. haemolyticus* *rseP* | This study |
| *Em^r^: Erythromycin resistant | | |

**Table S5. Bacteriocins used in this study**

| **Bacteriocins (*)** | | **Amino acid sequence** | **Reference** |
| --- | --- | --- | --- |
| K1 | | MKFKFNPTGTIVKKLTQYEIAWFKNKHGYYPWEIPRC | ^18^ |
| Ej97 | | MLAKIKAMIKKFPNPYTLAAKLTTYEINWYKQQYGRYPWERPVA | ^19^ |
| H1 | | MKFKFNPTGTIVKKLTQYEINWYKQQYGRYPWERPVA | This study |
| GakA*  GakB*  GakC* |  | MGAIIKAGAKIVGKGVLGGGASWLGWNVGEKIWK | ^20^  ^20^  ^20^ |
|  |  | MGAIIKAGAKIIGKGLLGGAAGGATYGGLKKIFG |  |
|  |  | MGAIIKAGAKIVGKGALTGGGVWLAEKLFGGK |  |
| Micrococcin P1** | | SCTTCVCTCSCCTT | ^21^ |
| * Components of garvicin KS; ** Micrococcin P1 has exstensive post-translational modifications | | | |

**Table S6 Primers used in this study**

| **Primer and category** | **Sequence (5’-3’)** |
| --- | --- |
| *RseP* amplification |  |
| SH_7076_4_21 _RseP_F | GGA GTA TGA TTC ATA TGA GCT ATT TAA TCA CTA TTG TCT CAT TT |
| SH_7076_4_21 _RseP_R | TCG AAC CCG GGG TAC CTT ACA AGA AAT AAC GTT GTA TAT CGT TC |
| Sequencing |  |
| SH_7076_4_21_RseP_Seq_F1 | TTG AGT GCA CAT TTG ACT AGA C |
| SH_7076_4_21_RseP_Seq_F2 | ATC GCT CCA CGA CAT CGA C |
| SH_7076_4_21_RseP_Seq_F3 | GAA CGA AAC TTT GTA TAC CAT CCG |
| SH_7076_4_21_RseP_Seq_R1 | ACT CAA TGC TTC TGC TTC AGC |
| SH_7076_4_21_RseP_Seq_R2 | GCT GCA GAC TGA ATG TCA TC |
| SH_7076_4_21_RseP_Seq_R3 | ATG TAC TGG CAC TAA CAA ACT G |
| SH_7076_4_21_RseP_Seq_R4 | AAA TTC GAC CAC CAT CAA GTG C |

##

Supplementary references

1 McShan, W. M. & Shankar, N. in *The Enterococci* 409-415 (2002).

2 Cintas, L. M. *et al.* Enterocins L50A and L50B, two novel bacteriocins from *Enterococcus faecium* L50, are related to staphylococcal hemolysins. *J Bacteriol* **180**, 1988-1994, doi:10.1128/JB.180.8.1988-1994.1998 (1998).

3 Cintas, L. M., Casaus, P., Havarstein, L. S., Hernandez, P. E. & Nes, I. F. Biochemical and genetic characterization of enterocin P, a novel sec-dependent bacteriocin from *Enterococcus faecium* P13 with a broad antimicrobial spectrum. *Appl Environ Microbiol* **63**, 4321-4330, doi:10.1128/AEM.63.11.4321-4330.1997 (1997).

4 Herranza C, P. C. P., Mukhopadhyaya S, Martı́nez JM, Rodrı́guez JM, Nes IF, Hernández PE, Cintas LM. *Enterococcus faecium* P21: a strain occurring naturally in dry-fermented sausages producing the class II bacteriocins enterocin A and enterocin B. *Food Microbiology* **18**, 115-131, doi:https://doi.org/10.1006/fmic.2000.0382 (2001).

5 Marekova, M., Laukova, A., Skaugen, M. & Nes, I. Isolation and characterization of a new bacteriocin, termed enterocin M, produced by environmental isolate *Enterococcus faecium* AL41. *J Ind Microbiol Biotechnol* **34**, 533-537, doi:10.1007/s10295-007-0226-4 (2007).

6 Petra S.Tichaczek, J.-M., Ingolf F.Nes, Rudi F.Vogel, Walter P.Hammes. Characterization of the Bacteriocins Curvacin A from Lactobacillus curvatus LTH1174 and Sakacin P from *L. sake* LTH673. *Systematic and Applied Microbiology* **15**, 460-468, doi:https://doi.org/10.1016/S0723-2020(11)80223-7 (1992).

7 Sobrino, O. J. *et al.* Sakacin M, a bacteriocin-like substance from *Lactobacillus sake* 148. *Int J Food Microbiol* **16**, 215-225, doi:10.1016/0168-1605(92)90082-e (1992).

8 Claesson, M. J. *et al.* Multireplicon genome architecture of *Lactobacillus salivarius*. *Proc Natl Acad Sci U S A* **103**, 6718-6723, doi:10.1073/pnas.0511060103 (2006).

9 Sanchez, J. *et al.* Antimicrobial and safety aspects, and biotechnological potential of bacteriocinogenic enterococci isolated from mallard ducks (Anas platyrhynchos). *Int J Food Microbiol* **117**, 295-305, doi:10.1016/j.ijfoodmicro.2007.04.012 (2007).

10 Bolotin, A. *et al.* The complete genome sequence of the lactic acid bacterium *Lactococcus lactis* ssp. lactis IL1403. *Genome Res* **11**, 731-753, doi:10.1101/gr.gr-1697r (2001).

11 Hechard, Y., Pelletier, C., Cenatiempo, Y. & Frere, J. Analysis of sigma(54)-dependent genes in *Enterococcus faecalis*: a mannose PTS permease (EII(Man)) is involved in sensitivity to a bacteriocin, mesentericin Y105. *Microbiology (Reading)* **147**, 1575-1580, doi:10.1099/00221287-147-6-1575 (2001).

12 Marciset, O., Jeronimus-Stratingh, M. C., Mollet, B. & Poolman, B. Thermophilin 13, a nontypical antilisterial poration complex bacteriocin, that functions without a receptor. *J Biol Chem* **272**, 14277-14284, doi:10.1074/jbc.272.22.14277 (1997).

13 Pain, M., Hjerde, E., Klingenberg, C. & Cavanagh, J. P. Comparative Genomic Analysis of *Staphylococcus haemolyticus* Reveals Key to Hospital Adaptation and Pathogenicity. *Front Microbiol* **10**, 2096, doi:10.3389/fmicb.2019.02096 (2019).

14 Cavanagh, J. P. *et al.* Whole-genome sequencing reveals clonal expansion of multiresistant *Staphylococcus haemolyticus* in European hospitals. *J Antimicrob Chemother* **69**, 2920-2927, doi:10.1093/jac/dku271 (2014).

15 Kleerebezem, M. *et al.* Complete genome sequence of *Lactobacillus plantarum* WCFS1. *Proc Natl Acad Sci U S A* **100**, 1990-1995, doi:10.1073/pnas.0337704100 (2003).

16 Sorvig, E. *et al.* Construction of vectors for inducible gene expression in *Lactobacillus sakei* and *L. plantarum*. *FEMS Microbiol Lett* **229**, 119-126, doi:10.1016/S0378-1097(03)00798-5 (2003).

17 Sorvig, E., Mathiesen, G., Naterstad, K., Eijsink, V. G. H. & Axelsson, L. High-level, inducible gene expression in *Lactobacillus sakei* and *Lactobacillus plantarum* using versatile expression vectors. *Microbiology (Reading)* **151**, 2439-2449, doi:10.1099/mic.0.28084-0 (2005).

18 Ovchinnikov, K. V. *et al.* The Leaderless Bacteriocin Enterocin K1 Is Highly Potent against *Enterococcus faecium*: A Study on Structure, Target Spectrum and Receptor. *Front Microbiol* **8**, 774, doi:10.3389/fmicb.2017.00774 (2017).

19 Frank, K. L. *et al.* Use of recombinase-based in vivo expression technology to characterize *Enterococcus faecalis* gene expression during infection identifies *in vivo*-expressed antisense RNAs and implicates the protease Eep in pathogenesis. *Infect Immun* **80**, 539-549, doi:10.1128/IAI.05964-11 (2012).

20 Ovchinnikov, K. V. *et al.* Novel Group of Leaderless Multipeptide Bacteriocins from Gram-Positive Bacteria. *Appl Environ Microbiol* **82**, 5216-5224, doi:10.1128/AEM.01094-16 (2016).

21 Ciufolini, M. A. & Lefranc, D. Micrococcin P1: structure, biology and synthesis. *Nat Prod Rep* **27**, 330-342, doi:10.1039/b919071f (2010).
